# Inhibition of autophagy by 3-MA enhances endoplasmic reticulum stress-induced apoptosis in human nasopharyngeal carcinoma cells

**DOI:** 10.3892/ol.2013.1498

**Published:** 2013-07-29

**Authors:** LELE SONG, HAO LIU, LINYAN MA, XUDNG ZHANG, ZHIWEN JIANG, CHENCHEN JIANG

**Affiliations:** 1 Faculty of Pharmacy, Bengbu Medical College, Anhui Engineering Technology Research Center of Biochemical Pharmaceuticals, Bengbu, Anhui 233030, P.R. China; 2 Department of Pharmacy, The First Affilated Hospital of Bengbu Medical College, Affilated Tumor Hospital of Bengbu Medical College, Bengbu, Anhui 233004, P.R. China; 3 Priority Research Center for Cancer Research, University of Newcastle, Callaghan, New South Wales 2308, Australia

**Keywords:** nasopharyngeal carcinoma, autophagy, apoptosis, endoplasmic reticulum stress, ionizing radiation, 3-methyladenine

## Abstract

Radiotherapy and adjuvant cisplatin chemotherapy are the mainstream treatments for nasopharyngeal carcinoma (NPC), which effectively improve the outcome and reduce tumor recurrence. However, the resistance mechanism(s) involved in radiotherapy and chemotherapy, which is the main barrier in NPC treatment, remains undefined. Therefore, there is an urgent requirement for the identification of new therapeutic strategies or adjuvant drugs. In the present study, the effects of autophagy inhibitors on endoplasmic reticulum (ER) stress-induced autophagy was investigated. Combining 3-methyladenine (3-MA) with cisplatin (DDP), ionizing radiation (IR), 2-deoxy-D-glucose (2-DG) or tunicamycin (TM) resulted in enhanced cell death, as revealed by MTT and colony formation assays. Flow cytometry results demonstrated that the sensitivity of NPC cells to DDP- and IR-induced apoptosis was not significant. DDP, IR, 2-DG and TM induced ER stress and autophagy. Using fluorescence microscopy, 3-MA was identified to increase the apoptotic cell death induced by DDP, IR, 2-DG or TM. In addition, 3-MA inhibited the increased autophagy induced by DDP, IR, 2-DG or TM, as demonstrated by western blot analysis and immunocytochemistry results. Results of the present study indicate that autophagy acts as a protective mechanism response to the apoptosis induced by DDP, IR, 2-DG or TM.

## Introduction

Inappropriate activation of survival signaling pathways causes uncontrolled proliferation, resistance to apoptosis and an increased motility of cells and is important for cancer development, progression and resistance to treatment (1). In nasopharyngeal carcinoma (NPC), standard treatments with systemic agents, including adjuvant cisplatin chemotherapy, has been unrewarding in general (2,3), which is primarily due to the failure in the induction of cell death and the development of the resistance mechanism(s) to chemotherapeutic reagents (4,5) and radioactive rays (6).

Apoptosis is the principal mechanism by which cells are physiologically eliminated in metazoan organisms (7). During apoptotic death, cells are digested by caspases and packaged into apoptotic bodies as a mechanism to avoid immune activation. Necrosis, previously hypothesized as a passive, unorganized method of cell death, has emerged as an alternate form of programmed cell death whose activation may have important biological consequences, including the induction of an inflammatory response (7). Autophagy has also been reported as a possible mechanism for non-apoptotic death despite evidence from a number of species showing that autophagy represents a survival strategy in times of stress. Recent advances have been important for the definition of the function and mechanism of programmed necrosis and the role of autophagy in cell survival and suicide (7).

Autophagy is a highly-conserved pathway in eukaryotic cells that has evolved to degrade bulk cytoplasmic material (8). Autophagy, a process of ‘self-eating’, has been classically studied in response to energy deprivation, including that which results from nutritional starvation of amino acids or fatty acids (9). Autophagy also provides an important function in the clearance of aggregated or misfolded proteins (10). However, it has also been reported to play a dual role in cancer and the induction of autophagy has been demonstrated to inhibit tumor cell growth and result in autophagic cell death (11). By contrast, it has been shown that autophagy may function as a cytoprotective mechanism by responding to stress situations, including hypoxia, low energy, oxidative stress and damaged mitochondria (12).

In the present study, we aimed to investigate the role of 3-methyladenine (3-MA) in NPC therapies, including chemotherapy and radiotherapy, as well as determine whether cisplatin (DDP), ionizing radiation (IR), 2-deoxy-D-glucose (2-DG) or tunicamycin (TM) induce autophagy via endoplasmic reticulum (ER) stress.

## Materials and methods

### Cell lines

Human NPC cell lines, CNE-1 and CNE-2, were obtained from the American Type Culture Collection (Manassas, VA, USA) and maintained at the Anhui Engineering Technology Research Center of Biochemical Pharmaceuticals (Anhui, China). Cells were cultured in DMEM medium containing 10% NBCS (both Gibco-BRL, Carlsbad, CA, USA), 2 mmol/l L-glutamine, 100 U/ml penicillin and 100 μg/ml streptomycin and cultured at 37°C in a humidified atmosphere of 95% air and 5% CO_2_.

### Drugs and antibodies

DDP was purchased from Qilu Pharmaceutical Co., Ltd. (Jinan, China). 2-DG and TM were purchased from Sigma-Aldrich (St. Louis, MO, USA). 3-MA was purchased from an affiliate of Merck KgaA (Darmstadt, Germany). Horseradish peroxidase (HRP)-conjugated anti-rabbit IgG, anti-rabbit IgG (H+L chains)-fluorescein isothiocyanate (FITC), anti-LC3 and anti-beclin 1 were purchased from MBL Biotech Co. (Beijing, China). β-actin was obtained from Santa Cruz Biotechnology (Santa Cruz, CA, USA).

### Cell proliferation assay

3-(4,5-Dimethylthiazol-2-yl)-2,5-di- phenyltetrazolium bromide (MTT)was performed as described previously (13). Briefly, cells were plated in 96-well culture clusters (Costar, Cambridge, MA, USA) at a density of 1×10^5^ cells/well in triplicate. MTT (5.0 mg/l; 15 μl) was added 4 h later, followed by the addition of 150 μl DMSO into each well. The absorbance (A) of the formazan product was determined at 570 nm using a plate microreader and calculated using the following formula: Cell viability (%) = (A_sample_ − A_blank_)/(A_control_ − A_blank_) × 100.

### Annexin V/propidium iodide (PI)-FITC double staining

Cell apoptosis was detected using the annexin V-FITC apoptosis detection kit (BD Pharmingen, San Diago, CA, USA) according to the manufacturer’s instructions. Briefly, the cells were seeded in 24-well plates and then subjected to various experimental conditions for 24 h. Following incubation, the cells were trypsinized and suspended in 1 ml phosphate buffered saline (PBS). In each cell suspension, 2×10^5^ cells were centrifuged and re-suspended in 500 μl 1X binding buffer. In each sample, 5 μl annexin V-FITC and 5 μl PI were added. The mixture was incubated for 5 min in the dark and immediately analyzed using a FACSCalibur flow cytometer and Cell Quest software (BD Biosciences, San Jose, CA, USA).

### Colony formation assay

A colony formation assay was performed as described previously (14). In brief, the cells were seeded at 1×10^3^ cells/well in 6-well culture plates, allowed to grow for 24 h and then treated as indicated. The cells were then washed twice with ice-cold PBS and fixed with ice-cold methanol for 10 min. Methanol was aspirated off from the plates, 0.5% crystal violet solution (Sciencelab.com, Inc., Houston, TX, USA) was added and the plates were incubated at room temperature for 10 min. Distilled water was used to rinse the plate. Images were captured with the Bio-Rad VersaDoc™ imaging system (Hercules, CA, USA).

### Western blot analysis

A western blot analysis was performed as described previously (15). Labeled bands were detected by the Immun-Star™ HRP Chemiluminescence kit (Bio-Rad) and images were captured using the Bio-Rad VersaDoc image system.

### Immunocytochemistry

The cells were seeded in 12-well plates at a density of 1.2×10^5^ cells/well. Following 24 h of drug or IR exposure, the cells were washed with PBS twice and fixed with PBS containing 4% paraformaldehyde for 15 min at room temperature. Subsequent to being blocked with 5% bovine serum albumin for 2 h, the cells were washed with PBS and incubated with anti-beclin 1 antibody (1:100) overnight at 4°C. The cells were then washed with PBS and incubated in the dark with 100 μl FITC-conjugated anti-rabbit IgG (1:100) for 2 h. The cells were washed with PBS and the fluorescence signal was detected by an inverted fluorescent microscope (Olympus Corporation, Tokyo, Japan).

### DAPI staining

The cells were seeded in 12-well plates at a density of 1.2×10^5^ cells/well. Following 24 h of drug or IR exposure, the cells were washed with PBS twice and fixed with PBS containing 4% PFA for 15 min at room temperature. The cells were washed further with PBS and then stained with 10 μg/ml DAPI (Beyotime Biotech, Jiangsu, China) for 5 min in the dark at room temperature. The solution was then removed and the cells were washed twice with PBS and analyzed using an inverted fluorescence microscope.

### Mitochondrial membrane potential (Δψm)

The cells were seeded at 1×10^5^ cells/well in 24-well plates and allowed to culture to reach exponential growth for 24 h prior to treatment. Changes in Δψm were studied by staining the cells with the cationic dye, JC-1, according to the manufacturer’s instructions (Molecular Probes, Eugene, OR, USA), as described previously (16). CCCP, which induces apoptosis, was used as the positive control.

### Statistical analysis

All experiments described were performed at least in triplicate. Data are expressed as mean ± SD. All statistical analyses were performed using two-tailed paired Student’s t-tests. P<0.05 was considered to indicate a statistically significant difference.

## Results

### Sensitivity of human NPC cells to DDP- and IR-induced apoptosis is not significant

**Figure 1:**
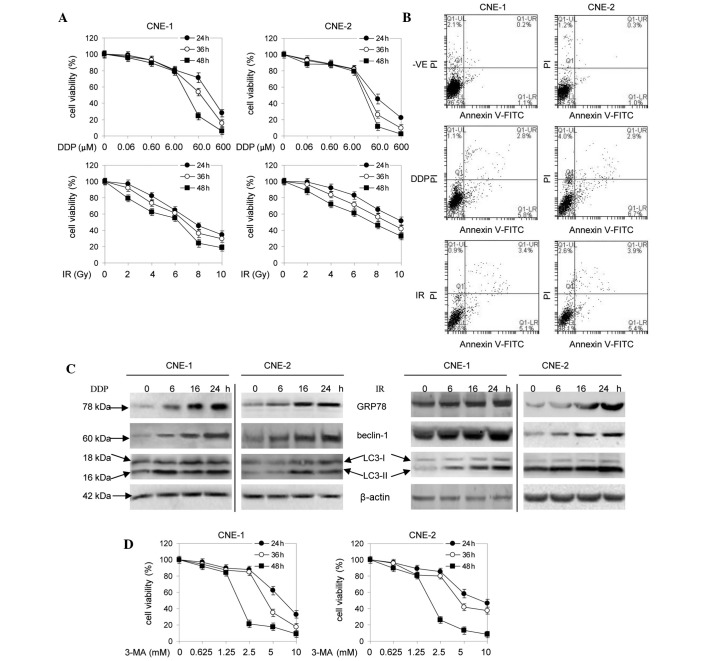
Effects of DDP, IR or 3-MA on the viability and apoptosis of NPC cells. (A) Cells were treated with 0–600 μmol/l DDP or 0–10 Gy of IR for 24, 36 or 48 h and viability was measured by MTT assay. (B) Cells were treated with 6 μmol/l DDP or 4 Gy IR for 24 h and cell death was detected by Annexin V-FITC/PI. (C) Cells were treated with 6 μmol/l DDP or 4 Gy IR for 6, 16 and 24 h. (D) Cells were treated with 0–10 mmol/l of 3-MA for 24, 36 or 48 h and viability was measured by MTT assay (n=3). DDP, cisplatin; IR, ionizing radiation, 3-MA, 3-methyladenine; NPC, nasopharyngeal carcinoma; MTT, 3-(4,5-dimethylthiazol-2-yl)-2,5-di-phenyltetrazolium bromide; FITC, fluorescein isothiocyanate; PI, propidium iodide; LC3, microtubule-associated protein 1 light chain 3.

The proliferation of the NPC cells was inhibited by various concentrations of DDP and IR (Fig. 1A). This inhibition was enhanced with increasing concentrations of DDP and IR and prolonged with increasing duration of exposure of the cells to DDP and IR. Following treatment with 6.00 μmol/l DDP for 24, 36 and 48 h, the survival rate of the CNE-1 and CNE-2 cells reached 80.74, 79.95 and 78.92% and 82.15, 82.05 and 79.13%, respectively. Treatment of the CNE-1 and CNE-2 cells with 4 Gy IR for 24, 36 and 48 h resulted in a survival rate of 2.29, 73.51 and 62.35% and 92.35, 83.58 and 72.37%, respectively. Based on these results, 6.00 μmol/l DDP and 4 Gy IR was used, each for 24 h, for further experiments.

Following treatment with 6.00 μmol/l DDP for 24 h, the induction of apoptosis in the CNE-1 and CNE-2 cells was only 5.8 and 6.7%, respectively, which was not statistically significant when compared with the negative control group (Fig. 1B). Subsequent to treatment with 4 Gy IR for 24 h, the induction of apoptosis in the CNE-1 and CNE-2 cells was only 5.1 and 5.4%, respectively, which was not statistically significant when compared with the negative control group (Fig. 1B).

### DDP and IR induce ER stress and autophagy in human NPC cells

It has been previously demonstrated that DDP and IR activate autophagy via ER stress (17). Therefore, the expression levels of GRP78, LC-3 and beclin 1 were determined. The levels of GRP78 and beclin 1 were increased in the cells treated with 6.00 μmol/l DDP or 4 Gy IR at various times (Fig. 1C). Western blot analysis revealed that microtubule-associated protein 1 light chain 3 (LC3) was converted from the free form (LC3-I) to a lipid-conjugated membrane-bound form (LC3-II) in the cells treated with 6.00 μmol/l DDP or 4 Gy IR for 24 h (Fig. 1C). As hypothesized, these observations indicate that DDP or IR induces ER stress and autophagy in human NPC cells.

### 3-MA enhances the sensitivity of human NPC cells to DDP and IR

**Figure 2:**
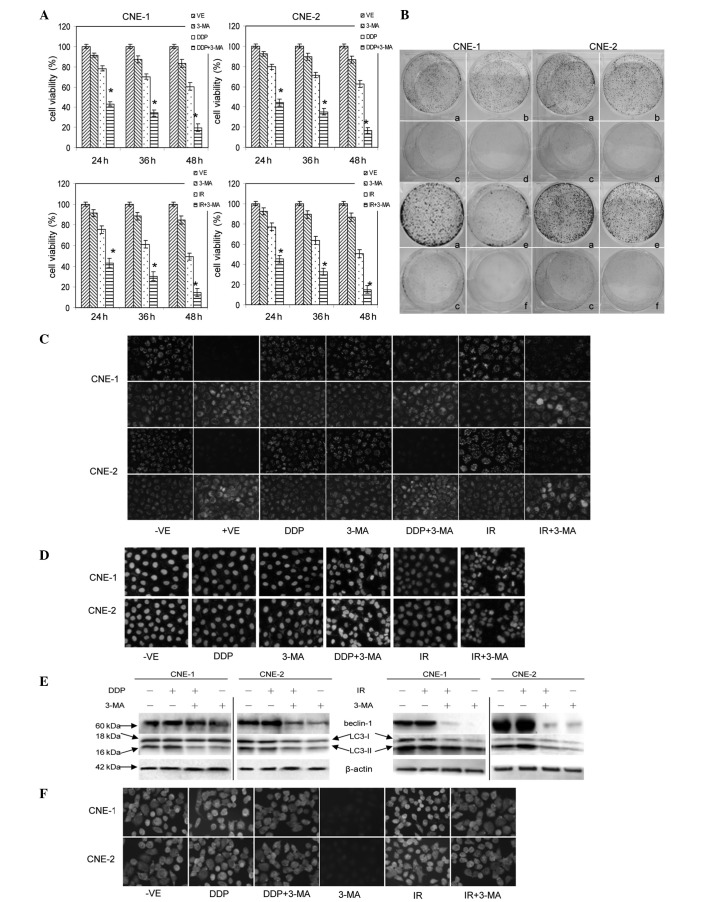
Effects of 3-MA with DDP or IR on the viability, apoptosis and autophagy of cultured NPC cells. (A) Cells were treated with 1 mmol/l 3-MA combined with 6 μmol/l DDP or 4 Gy IR for 24, 36 or 48 h and viability was measured by MTT assay. ^*^P<0.05 vs. control group. (B) Cells were treated with (a) fresh culture medium, (b) 0.6 μmol/1 DDP or (e) 0.4 Gy IR, (c) 0.1 mmol/l 3-MA and (d) 0.6 μmol/l DDP or (f) 0.4 Gy IR combined with 0.1 mmol/l 3-MA for 5 days. Cells were treated with 6 μmol/l DDP or 4 Gy IR in the presence or absence of 1 mmol/l 3-MA for 24 h. (C) Cells were collected for staining with JC-1; (D) cells were collected for staining with DAPI; (E) cells were subjected to western blot analysis; and (F) cells were collected for staining with anti-beclin 1 antibody (n=3). DDP, cisplatin; IR, ionizing radiation, 3-MA, 3-methyladenine; NPC, nasopharyngeal carcinoma; MTT, 3-(4,5-dimethylthiazol-2-yl)-2,5-di-phenyltetrazolium bromide; LC3, microtubule-associated protein 1 light chain 3.

The proliferation of the NPC cells was inhibited by various concentrations of 3-MA (P<0.05; Fig. 1D). Inhibition was enhanced with increasing concentrations of 3-MA and prolonged with increasing duration of cell exposure to 3-MA. Combining 1 mmol/l 3-MA with 6.00 μmol/l DDP or 4 Gy IR reduced cell viability compared with the individual use of each agent, as revealed by MTT assay (Fig. 2A). Colony formation assays were used to further confirm that 3-MA enhanced the sensitivity of the NPC cells to DDP or IR. The two cell lines formed fewer colonies when treated with 3-MA (0.1 mmol/l) in the presence of DDP (0.60 μmol/l) or IR (0.4 Gy) compared with the individual agent (Fig. 2B).

JC-1 staining assay results reveal the conversion of fluorescence from red to green in the cells treated with 50 μmol/l CCCP for 20 min. Following treatment with 1 mmol/l 3-MA combined with 6.00 μmol/l DDP or 4 Gy IR for 24 h, the extent of the conversion of fluorescence from red to green in the cells was significant (Fig. 2C). These observations indicate that 3-MA promotes apoptosis in DDP- or IR-treated NPC cells. Consistent with this, the DAPI staining results in Fig. 2D revealed typical morphological changes, including chromatin condensation and apoptotic body formation, in the NPC cells treated with 3-MA combined with DDP or IR. By contrast, the cells in the control group did not reveal any abnormal morphologies. These results indicate that 3-MA promotes apoptosis in IR- or DDP-treated NPC cells.

### Effects of 3-MA with DDP or IR on autophagy in human NPC cells

The GRP78 and beclin 1 protein levels were increased in the cells treated with 6.00 μmol/l DDP or 4 Gy IR for 24 h. Western blot analysis revealed the conversion of LC3 from LC3-I to LC3-II in the cells treated with 6.00 μmol/l DDP or 4 Gy IR for 24 h (Fig. 2E). Notably, the treatment with 1 mmol/l 3-MA reversed these effects. The results from the immunocytochemistry analysis of the expression of beclin 1 further confirm these observations (Fig. 2F).

### Effects of 3-MA with 2-DG or TM on the proliferation and apoptosis of human NPC cells

**Figure 3:**
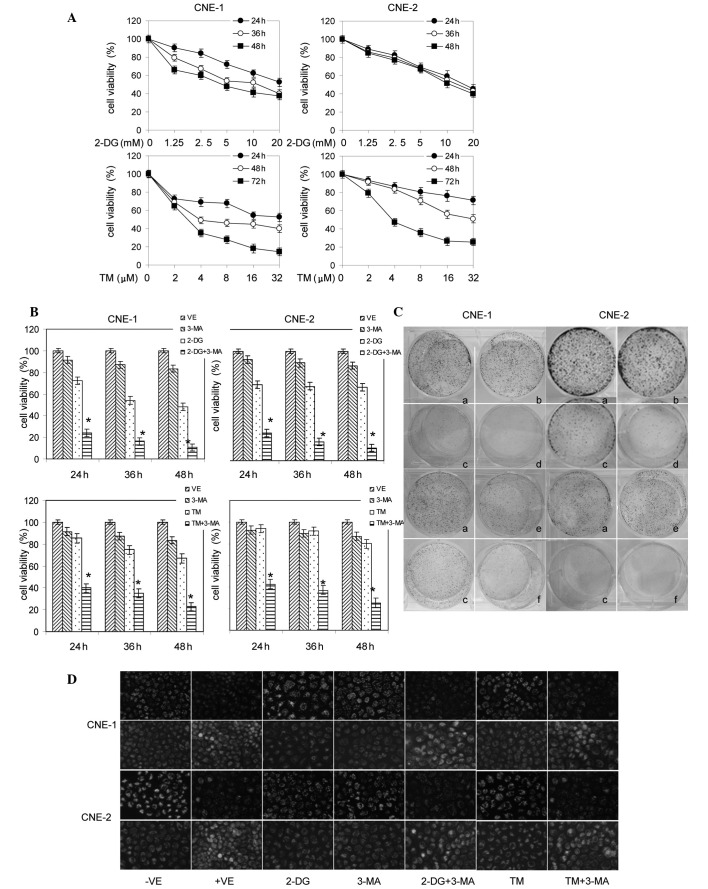
Effects of 3-MA with 2-DG or TM on the viability and apoptosis of cultured NPC cells. (A) Cells were treated with various concentrations (0–20 mmol/l) of 2-DG or (0–32 μmol/l) of TM for 24, 36 and 48 h and cell viability was measured by MTT assay. (B) Cells were treated with 1mmol/l 3-MA combined with 5 mmol/l 2-DG or 1 μmol/l TM for 24, 36 and 48 h and cell viability was measured by MTT assay. ^*^P<0.05 vs. control group. (C) Cells were treated with (a) fresh culture medium, (b) 0.5 mmol/l 2-DG or (e) 0.1 μmol/l TM, (c) 0.1 mmol/l 3-MA and (d) 0.5 mmol/l 2-DG or (f) 0.1 μmol/l TM combined with 0.1 mmol/l 3-MA for 5 days. (D) Cells were treated with 5 mmol/l 2-DG or 1 μmol/l TM in the presence or absence of 1 mmol/l 3-MA for 24 h. Cells were collected for staining with JC-1 (n=3). 3-MA, 3-methyladenine; 2-DG, 2-deoxy-D-glucose; TM, tunicamycin; NPC, nasopharyngeal carcinoma; MTT, 3-(4,5-dimethylthiazol-2-yl)-2,5-di-phenyltetrazolium bromide.

The proliferation of the NPC cells was inhibited by various concentrations of 2-DG or TM (Fig. 3A). This inhibition was enhanced with increasing concentrations of 2-DG or TM, and prolonged with the increasing duration of cell exposure to 2-DG or TM. Following treatment with 5 mmol/l 2-DG for 24, 36 and 48 h, the survival rate of the CNE-1 and CNE-2 cells reached 72.13, 53.14 and 47.99% and 69.32, 68.02 and 67.02%, respectively. When the cells were treated with 2 μmol/l TM for 24, 48 and 72 h, the survival rate of the CNE-1 and CNE-2 cells reached 72.43, 69.05 and 65.13% and 93.28, 91.58 and 79.21%, respectively. Based on these results, 5 mmol/l 2-DG and 1 μmol/l TM were each used for 24 h for further experiments.

The treatment of the cells with 1 mmol/l 3-MA combined with 5 mmol/l 2-DG or 1 μmol/l TM reduced the cell viability compared with the individual use of each agent, as revealed by MTT assay (P<0.05; Fig. 3B). These results were further supported by the colony formation assays (Fig. 3C).

Colony formation assays were used to further confirm the fact that 3-MA enhances the sensitivity of human NPC cells to 2-DG and TM. The two cell lines formed fewer colonies when treated with 3-MA in the presence of 2-DG or TM compared with with 2-DG or TM alone.

**Figure 4:**
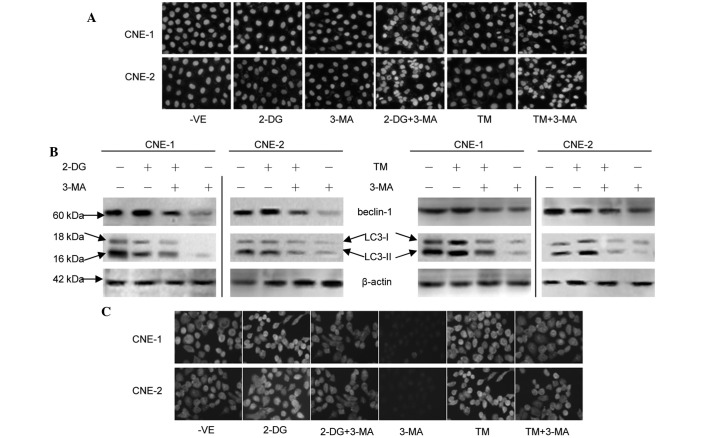
Effects of 3-MA with 2-DG or TM on the apoptosis and autophagy of NPC cells. (A) Cells were treated with 6 μmol/l DDP or 4 Gy IR in the presence or absence of 1 mmol/l 3-MA for 24 h. Cells were collected for staining with DAPI. (B) Cells were treated with 5 mmol/l 2-DG or 1 μmol/l TM in the presence or absence of 1 mmol/l 3-MA for 24 h. (C) Cells were treated with 5 mmol/l 2-DG or 1 μmol/l TM in the presence or absence of 1 mmol/l 3-MA for 24 h. Cells were collected for staining with anti-beclin 1 antibody (n=3). 3-MA, 3-methyladenine; 2-DG, 2-deoxy-D-glucose; TM, tunicamycin; NPC, nasopharyngeal carcinoma; DDP, cisplatin; MTT, 3-(4,5-dimethylthiazol-2-yl)-2,5-di-phenyltetrazolium bromide.

The treatment of the cells with 1 mmol/l 3-MA combined with 5 mmol/l 2-DG or 1 μmol/l TM for 24 h resulted in the significant conversion of fluorescence from red to green in the cells (Fig. 3D). This indicates that 3-MA promotes apoptosis in 2-DG- or TM-treated NPC cells. These data were further supported by the DAPI staining results. Fig. 4A demonstrates that the treatment of the cells with 2-DG or TM did not appreciably induce apoptosis in the cells, but typical morphological changes associated with apoptosis, including chromatin condensation, apoptotic body formation and DNA fragmentation, were evident in the cells treated with 3-MA combined with 2-DG or TM. By contrast, the cells in the control group did not exhibit any abnormal morphology.

The beclin 1 protein levels were increased in the cells treated with 5 mmol/l 2-DG or 1 μmol/l TM for 24 h (Fig. 4B). Western blot analysis revealed the conversion of LC3 from LC3-I to LC3-II in the cells treated with 5 mmol/l 2-DG or 1 μmol/l TM for 24 h. Notably, 1 mmol/l 3-MA was able to reverse this effect. The results from the immunocytochemistry analysis of the expression of beclin 1 further confirmed these observations (Fig. 4C).

## Discussion

The 5-year survival rate following the combination of radiotherapy and adjuvant DDP chemotherapy is only 50–60% and the rates of 5-year cumulative local relapse and distant metastasis are 20–30 and 20–25%, respectively (17). Etiological factors that have been identified for NPC include Epstein-Barr virus infection, environmental risk factors and genetic susceptibility (18).

In the present study, DDP or IR was shown to induce cell death. However, the sensitivity of human NPC cells to DDP and IR-induced apoptosis was not significant.

Several studies have reported that ER stress induces autophagy in mammalian cancer cell lines and mouse embryonic fibroblasts (19,20). ER stress is caused by the accumulation of misfolded or premature proteins in the ER lumen or the cytosol. Changes in the environment of the ER lumen, including changes in calcium levels, redox status and ER function, all affect correct protein folding. The major mitigating mechanism for ER stress is the unfolded protein response (UPR), which is mediated by several signaling mechanisms that alleviate ER stress. In mammalian cells, UPR is mediated by the PERK, ATF6 and IRE1 pathways. Current studies support the hypothesis that an ER chaperone protein, BiP (also known as GRP78), serves as a master UPR regulator and plays essential roles in activating IRE1, PERK and ATF6 in response to ER stress (7,13). As demonstrated in yeast and mammalian cells (9,11), ER stress activates other major cellular degradation mechanisms, including autophagy, which, in turn, affects ER stress and, consequently, cell death.

3-MA is a popular inhibitor of the autophagic pathway (20). 3-MA has been reported to inhibit the activity of PI3-kinase and to block the formation of preautophagosomes, autophagosomes and autophagic vacuoles. Autophagy has been reported to increase as a result of chemotherapy, resulting in the autophagic cell death of cancer cells (programmed cell death) or the adaptation of cancer cells to cytotoxicity induced by drugs, including in apoptosis (20). However, although autophagy has been hypothesized to represent a potential therapeutic target in adjuvant chemotherapy, the exact role and relevance of autophagy, autophagic cell death and apoptosis in cancer remains poorly understood and appears to be more complex than previously considered.

In the present study, 3-MA in combination with DDP or IR was shown to increase cell death more markedly than using DDP or IR alone. As demonstrated in Fig. 2C, 3-MA promoted apoptosis in the DDP- or IR-treated NPC cells. These data were further supported by the results of DAPI staining. Taken together, these results show that 3-MA enhances the sensitivity of NPC cells to DDP or IR. The western blot analysis revealed an increase in the GRP78 and beclin 1 protein levels, and the conversion of LC3 from LC3-I to LC3-II in the cells treated with DDP or IR. Notably, 3-MA was able to reverse this effect. These data were further supported by the immunocytochemistry analysis of beclin 1 expression. These observations indicate the important role of autophagy in ER stress-induced apoptosis.

In the current study, other ER stress inducers were used to demonstrate the broad applicability of our observations. The cells were exposed to the classic ER stress inducers, 2-DG and TM. 2-DG, a synthetic glucose analog that acts as a glycolytic inhibitor, is currently under clinical evaluation for targeting tumor cells. The glucosamine-containing nucleoside antibiotic, TM, is an inhibitor of N-linked glycosylation and of the formation of N-glycosidic protein-carbohydrate linkages.

The results from the MTT and colony formation assays demonstrated that the proliferation of the cells was inhibited by 2-DG and TM. 3-MA combined with 2-DG or TM reduced the cell viability compared with the individual use of each agent. These data were further supported by the results from the colony formation assay. The JC-1 staining assay revealed that 3-MA promotes apoptosis in the 2-DG- or TM-induced NPC cells. These data were further supported by the DAPI staining results.

Next, the molecular changes that occur following 2-DG or TM treatment were explored by immunoblotting assay. The beclin 1 protein levels increased following treatment with 2-DG or TM. Western blot analysis revealed the conversion of LC3 from LC3-I to LC3-II following treatment with 2-DG or TM. Notably, treatment with 3-MA reversed these effects. Finally, results from the immunocytochemistry analysis of the expression of beclin 1 further confirm these observations

The results of the present study indicate that autophagy is a protective mechanism in response to the apoptosis induced by DDP, IR, 2-DG or TM. These observations are likely to serve as a foundation for further investigations into autophagy in NPC. The extension of this study into *in vivo* studies and clinical trials is important.

## Abbreviations

NPC: nasopharyngeal carcinoma
DDP: cisplatin
IR: ionizing radiation
2-DG: 2-deoxy-D-glucose
TM: tunicamycin
3-MA: 3-methyladenine
ER: endoplasmic reticulum
GRP78: glucose-regulated protein 78
UPR: unfolded protein response
PERK: protein kinase-like ER kinase
ATF6: activation of transcription factor 6
IRE1: inositol-requiring transmembrane kinase and endonuclease 1
